# Is evolution Darwinian or/and Lamarckian?

**DOI:** 10.1186/1745-6150-4-42

**Published:** 2009-11-11

**Authors:** Eugene V Koonin, Yuri I Wolf

**Affiliations:** 1 National Center for Biotechnology Information, National Library of Medicine, National Institutes of Health, Bethesda, MD 20894, USA

## Abstract

**Background:**

The year 2009 is the 200^th ^anniversary of the publication of Jean-Bapteste Lamarck's *Philosophie Zoologique *and the 150^th ^anniversary of Charles Darwin's *On the Origin of Species*. Lamarck believed that evolution is driven primarily by non-randomly acquired, beneficial phenotypic changes, in particular, those directly affected by the use of organs, which Lamarck believed to be inheritable. In contrast, Darwin assigned a greater importance to random, undirected change that provided material for natural selection.

**The concept:**

The classic Lamarckian scheme appears untenable owing to the non-existence of mechanisms for direct reverse engineering of adaptive phenotypic characters acquired by an individual during its life span into the genome. However, various evolutionary phenomena that came to fore in the last few years, seem to fit a more broadly interpreted (quasi)Lamarckian paradigm. The prokaryotic CRISPR-Cas system of defense against mobile elements seems to function via a bona fide Lamarckian mechanism, namely, by integrating small segments of viral or plasmid DNA into specific loci in the host prokaryote genome and then utilizing the respective transcripts to destroy the cognate mobile element DNA (or RNA). A similar principle seems to be employed in the piRNA branch of RNA interference which is involved in defense against transposable elements in the animal germ line. Horizontal gene transfer (HGT), a dominant evolutionary process, at least, in prokaryotes, appears to be a form of (quasi)Lamarckian inheritance. The rate of HGT and the nature of acquired genes depend on the environment of the recipient organism and, in some cases, the transferred genes confer a selective advantage for growth in that environment, meeting the Lamarckian criteria. Various forms of stress-induced mutagenesis are tightly regulated and comprise a universal adaptive response to environmental stress in cellular life forms. Stress-induced mutagenesis can be construed as a quasi-Lamarckian phenomenon because the induced genomic changes, although random, are triggered by environmental factors and are beneficial to the organism.

**Conclusion:**

Both Darwinian and Lamarckian modalities of evolution appear to be important, and reflect different aspects of the interaction between populations and the environment.

**Reviewers:**

this article was reviewed by Juergen Brosius, Valerian Dolja, and Martijn Huynen. For complete reports, see the **Reviewers' reports **section.

## 

So who is that knight fighting for the honor of Nature?

Why - of course, it's the fiery Lamarck!

Osip Mandelshtam, Lamarck (1931)

(translated from Russian by EVK)

## Background

The celebrations of Darwin's 200 years jubilee and the 150 anniversary of *On the Origin of Species *[1] in 2009, to a large extent, overshadowed another anniversary: Jean-Bapteste Lamarck's magnum opus, *Philosophie Zoologique *[2], was published in 1809, the year of Darwin's birth [3]. Arguably, Lamarck's book was the first published manifesto of biological evolution as fittingly pronounced by Darwin himself in the later editions of the Origin [4-6]. Lamarck's concept of evolution was limited in scope: in particular, he did not believe in extinction of species but rather thought that species are gradually transformed into other species via phyletic modification. Lamarck also believed in the innate tendency of organisms to progress toward perfection down the succession of generations. In line with this idea, Lamarck speculated on an extremely simple and straightforward mechanism of evolutionary change whereby the use of a particular organ would lead to its gradual functional improvement that would be passed through generations (the example of the giraffe's neck is, probably, one of the most notorious "just so stories" in the history of biology). Later, a generalization of Lamarck's hypothetical mechanism became known as inheritance of acquired characters (characteristics) (IAC) to emphasize a key aspect of this mechanism, namely, the direct feedback between phenotypic changes and the (what is now known as) the genotype (genome). However, it should be stressed that the phrase "inheritance of acquired characters" is substantially imprecise in that Lamarck and his followers were very particular about adaptive (beneficial, useful) not just any acquired traits being inherited. Furthermore, inheritance of acquired characters certainly is not Lamarck's original idea; rather, it appears to have been "folk wisdom" in Lamarck's day [7]. Hereinafter we use the acronym IAC with this implicit understanding.

As already mentioned Darwin was well aware of Lamarck's work and generously acknowledged Lamarck's contribution in the chapter on his scientific forerunners that he included in the Origin starting with the 3^rd ^edition [4]. Darwin's own views on IAC markedly evolved. In the first edition of the Origin, he allowed IAC as a relatively unimportant mechanism of evolutionary change that was viewed as subsidiary to random, undirected variation. However, in the subsequent additions, Darwin viewed IAC as being progressively more consequential, apparently, in the face of the (in)famous Jenkin's nightmare of blending inheritance [8] which Darwin was unable to refute with a plausible mechanism of heredity. Even in Darwin's day, many scientists considered his giving in to Lamarckian inheritance a sign of weakness and a mistake.

In the 1880s, the renown German biologist August Weismann, in the context of his theory of germ plasm and germline-soma barrier, set out to directly falsify IAC in a series of experiments that became as famous as Lamarck's giraffe [9]. Almost needless to say, cutting off tails of Weismann's experimental rats not just failed to produce any tail-less pups but did not result in any shortening of the tail of the progeny whatsoever. Weismann's experiments delivered a serious blow to the public perception of IAC although, technically, they may be considered irrelevant to Lamarck's concept that, as already mentioned, insisted on the inheritance of beneficial changes, primarily, caused by use of organs, not senseless mutilation (which was generally known to have no effect on progeny long before Weismann, for instance, in the case of human circumcision, although claims to the contrary were common enough in Weismann's day and were the direct incentive for his experiments). Lamarck's ideas survived Weismann's experiments and more, perhaps, owing to the notion of the innate trend toward progress as a driving force of evolution that was attractive to various kinds of thinkers (and many individuals who hardly met that classification). Be it as it may, the fate of "Lamarckism" was arguably far worse than a quiet demise under the tails of Weismann's rats.

Inspired by ideas of progress in biological evolution, the flamboyant Viennese researcher and popularizer of science Paul Kammerer in the beginning of the 20^th ^century embarked on a two-decade long quest to prove IAC [10-14]. Kammerer's work included mostly experiments with amphibians that changed their color patterns and breeding habits depending on the environmental factors such as temperature and humidity. Strikingly, Kammerer insisted that the induced changes he observed were fully inheritable. Kammerer's experiments drew criticism due to his sloppy documentation and suspicious, apparently, doctored drawings and photographs. Kammerer defended his conclusions energetically but in 1923 his career came to end after the famous geneticist William Bateson found that Kammerer's showcase midwife toad that supposedly acquired black mating pads, a trait that was passed to the progeny, was actually injected with black ink. Kammerer killed himself within two years after this disgraceful revelation. Whether or not Kammerer was a fraud in the worst sense of the word remains unclear; it is thought that he might have used ink to "augment" a color change that he actually observed, a scientific practice that was not approved of even then, let alone now, but a far cry from flagrant cheating. Kammerer's findings might find their explanation in hidden variation among his animals that, unbeknownst to him, became subject to selection [11] or, alternatively, in epigenetic inheritance [12-14]. Under the most charitable of explanations, Kammerer ran a seriously sloppy operation, even if he unknowingly stumbled over important phenomena. Regardless of the specifics, the widely publicized "affaire Kammerer" hardly improved the reputation of Lamarckian inheritance. The worst for Lamarck was yet to come.

In a cruel irony, Kammerer was warmly welcomed by the Bolshevik leaders of the Soviet Union and nearly ended up moving his laboratory to that country. Despite the striking successes of Russian genetics in the 1920s (suffice it to recall the names of Chetverikov and Vavilov), the party leaders cherished the ideas of fast, planned, no-nonsense improvement of nature, including human nature. So, when the general situation in the country gravitated toward mass terror and hunger around 1930, a suitable team was found, under the leadership of the agronomist Trofim Lysenko. Lysenko and his henchmen were not scientists at all, not by any stretch, but utterly shameless criminals who exploited the abnormal situation in the country to amass in their hands extraordinary power over Soviet scientific establishment and beyond. Lamarckian inheritance that Lysenkoists, not without a certain perverse cleverness (to the modern reader, with a distinctly Orwellian tint), touted as a "true Darwinian" mechanism of evolution, was the keystone of their "theory". They took Lamarck's idea to grotesque extremes by claiming, for instance, that cuckoos repeatedly emerged *de novo *from eggs of small birds as a particularly remarkable adaptation. In his later years, after he fell from power, Lysenko retained an experimental facility where he reportedly fed cows butter and chocolate in an attempt to produce a breed stably giving high-fat milk. Mostly, the Lysenkoist "science of true Darwinism" was not even fraudulent because its adepts often did not bother to fake any "experiments" but simply told their ideologically inspired tales. This could have been comical if not for the fact that many dissenters literally paid with their lives, whereas almost all research in biology in the Soviet Union was hampered for decades. There is no reason to discuss Lysenko any further here; detailed accounts have been published [15-17], and the proceedings of the infamous 1948 session of the Soviet Agricultural Academy, where genetics was officially banished, remain a fascinating even if harrowing read [18].

What concerns me here is that, quite understandably, the unfortunate saga of Lysenko made the very idea of a Lamarckian mechanism actually operating during evolution repulsive and unacceptable to most biologists. The IAC itself remains, effectively, a derogatory phrase and is presented as a grave error in judgment in otherwise admiring accounts of Lamarck's work [3]. However, an objective look at several routes of emergence and fixation of evolutionary change that surfaced in the genomic era reveals mechanisms that appear suspiciously Lamarckian or at least quasi-Lamarckian. In this article, we discuss these classes of genomic changes and arrive to the conclusion that some mechanisms of evolution that meet all Lamarckian criteria do exist whereas, in many other instances, there is no sharp distinction between "Lamarckian" and "Darwinian" scenarios, with the two representing different aspects of the interaction between organisms and their environment that shapes evolution. Throughout this discussion, we stick to actual changes occurring in genomes, leaving apart the separate, fascinating subject of epigenetic inheritance.

### The Lamarckian mode of evolution, its distinction from the Darwinian mode and the criteria for the identification of Lamarckian inheritance

Before turning to the wide range of phenomena that seem to display all or some features of the mechanism of evolution proposed by Lamarck, it is of course necessary to define the Lamarckian paradigm and the criteria an evolutionary process must satisfy to be considered Lamarckian. In doing so, we deliberately do not dwell on the differences between Lamarck's original views and the numerous subsequent (mis)representations, but rather try to distill the essence of what is commonly known as IAC and the Lamarckian mode of evolution.

Lamarck's concept of heredity, which is also one of the two cornerstones of his evolutionary synthesis, stands on two principles that he promoted to the status of fundamental laws in *Philosophie Zoologique *and other texts:

1) Use and disuse of organs

2) The inheritance of acquired characters.

Lamarck directly linked the 'use and disuse' clause to effects of the environment on the "habits" of an organism and, through the said habits, on the "shape and nature" of body parts; and, of course, he considered these environment-effected adaptive changes to be heritable. Wrote Lamarck: "...nature shows us in innumerable...instances the power of environment over habit and of habit over the shape, arrangement and proportions of the parts of animals" [2]. Thus, Lamarck's idea of heredity is based on the threefold causal chain: environment-habit-form. Lamarck insisted on the essentiality of change in habits as an intermediate between the environment and (inheritable) change of organismal form: "Whatever the environment may do, it does not work any direct modification whatever in the shape and organization of animals. But great alterations in the environment of animals lead to great alterations in their needs, and these alterations in their needs necessarily lead to others in their activities. Now if the new needs become permanent, the animals then adopt new habits that last as long as the needs that evoked them". Lamarck was not original in his belief in IAC that appeared to be the folk wisdom of his day. However, he was both more specific than others in spelling out the above causal chain and, more importantly, he made this scheme the foundation of the far more original concept of evolution [7].

The second foundation of Lamarck's evolutionary synthesis was his belief in the innate tendency toward increasing organizational complexity - or, simply, progress - that, in Lamarck's view, shaped biological evolution along with the IAC. Although Lamarck often used the phrase "pouvoir de la vie" to denote this fundamental tendency, his idea was completely materialistic, even mechanistic, as he attributed the trend toward progress to the motion of fluids in the animal body which would carve channels and cavities in soft tissues, and gradually lead to the evolution of increasing organizational complexity. For a good measure, to explain why simply organized life form persisted despite the progressive character of evolution, Lamarck maintained that spontaneous generation was a constant source of primitive organisms. The ideas of spontaneous generation and the innate tendency toward progress, especially, with its naïve mechanistic underpinning, are hopelessly obsolete. Whether or not there is an overall trend toward increasing complexity over the course of evolution of life, remains a legitimate subject of debate [19-22], but of course, even those researchers who advocate the existence of such a trend would not characterize it as an "innate tendency". In what follows, we address the much more relevant and interesting problem of the IAC and its contribution to the evolutionary process.

In terms compatible with modern genetics, Lamarck's scheme entails that

**Figure 1:**
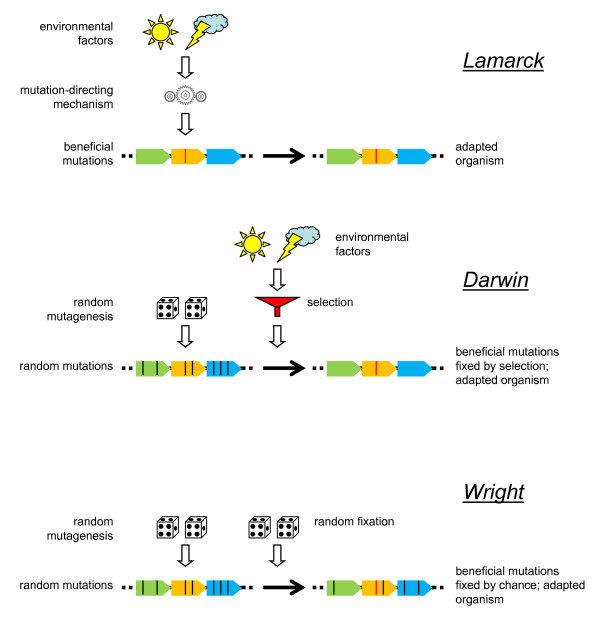
**Lamarckian, Darwinian, and Wrightian modalities of evolution**.

1) environmental factors cause genomic (heritable) changes

2) the induced changes (mutations) are targeted to a specific gene(s)

3) the induced changes provide adaptation to the original causative factor

(Figure 1). Obviously, the adaptive reaction to a specific environmental factor has to be mediated by a molecular mechanism that channels the genomic change. The distinction from the Darwinian route of evolution is straightforward: in the latter, the environment is not the causative agency but merely a selective force that may promote fixation of those random changes that are adaptive under the given conditions (Figure 1). The Darwinian scheme is simpler and less demanding than the Lamarckian one in that no specialized mechanisms are required to direct the change to the relevant genomic locus (loci) and restrict it to the specific modifications (mutations) providing the requisite adaptation. Indeed, it is the difficulty of discovering or even conceiving of mechanisms of directed adaptive change in genomes that have for decades relegated the Lamarckian scheme to trash heap of history. In the rest of this article we discuss the recent studies of several phenomena that seem to call for resurrection of the Lamarckian scenario of evolution. Of course, despite the substantial mechanistic differences, the Lamarckian and Darwinian schemes are similar in that both are essentially adaptive in the final outcome and in that regard are radically different from random drift (which may be denoted the "Wrightian modality of evolution", after Sewall Wright, the originator of the key concept of random genetic drift [23]) (Figure 1).

### Lamarckian and quasi-Lamarckian phenomena

#### The CRISPR-Cas system of antivirus immunity in prokaryotes: the showcase for a genuine Lamarckian mechanism

A recently discovered novel system of antiphage defense in archaea and bacteria seems to function via a straightforward Lamarckian mechanism. The system is known as CRISPR-Cas, where CRISPR stands for Clustered Regularly Interspaced Short Palindromic Repeats and Cas for CRISPR-associated genes (sometimes referred to as CASS or simply CRISPR system) [24-26]. The CRISPR are interspersed in the sense that they contain short unique inserts (spacers) embedded within each palindromic repeat unit. Archaeal and bacterial genomes contain cassettes of up to multiple CRIPSR units, in some cases, more than one cassette per genome. Although CRISPR have been recognized over 20 years ago, even before the first complete bacterial genome was sequenced, only much later was it realized that CRISPR cassettes are always adjacent in genomes to a group of *cas *genes that are predicted to encode various (predicted) enzymes involved in nucleic acid metabolism including several nucleases, a helicase, and a polymerase [27-29]. Serendipitously, it was discovered that some of the inserts in CRISPR cassettes are identical to fragments of bacteriophage and plasmid genes[30,31], so the hypothesis was formulated that the CRISPR-Cas system utilized the phage-derived sequences as guide molecules to destroy phage mRNAs analogously to the eukaryotic RNA interference (RNAi) [32]. Although most of the mechanistic details remain to be uncovered, the principal propositions of this hypothesis have been validated: the presence of an insert precisely complementary to a region of a phage genome is essential for resistance [33]; the guide RNAs form complexes with multiple Cas proteins and is employed to abrogate the infection [34-36]; and new inserts conferring resistance to cognate phages can be acquired [37,38]. An important modification to the original proposal is that, in the systems so far explored, the cleaved target is phage DNA itself rather than mRNA [39].

**Figure 2:**
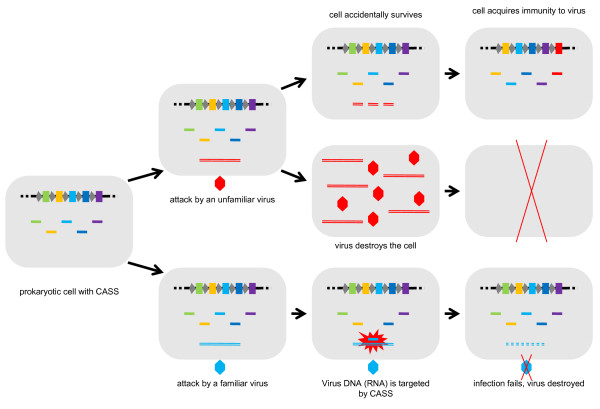
**The mechanism of CASS: a bona fide Lamarckian system**.

The mechanism of heredity and genome evolution embodied in the CRISPR-Cas system seems to be bona fide Lamarckian (Figure 2):

-an environmental cue (mobile element) is employed to directly modify the genome

-the resulting modification (unique, element-specific insert) directly affects the same cue that caused the modification

-the modification is clearly adaptive and is inherited by the progeny of the cell that encountered the mobile element.

A peculiarity of the CASS-mediated heredity is that it appears to be extremely short-lived: even closely related bacterial and archaeal genomes do not carry the same inserts, the implication being that, as soon as a bacterium or archaeon ceases to encounter a particular bacteriophage, the cognate insert rapidly deteriorates (indeed, the inserts hardly can be evolutionarily stable in the absence of strong selective pressure because a single mutation renders them useless)[32,37,38]. Nevertheless, the Lamarckian scenario seems undeniable in the case of CASS: adaptive evolution of organisms occurs directly in response to an environmental factor, the result being specific adaptation (resistance) to that particular factor [32].

#### Other potential Lamarckian systems functioning on the CASS principle

It is instructive to compare the hereditary and evolutionary features of the CASS with those of eukaryotic RNA interference (RNAi) and, more specifically, siRNA and piRNA, and immune systems, the two systems in eukaryotes that, at least, in general terms are functionally analogous to CASS. Neither of these systems seems to utilize a straightforward Lamarckian mechanism. Nevertheless, both can be considered to display certain "Lamarckian-like" features. The siRNA system (a distinct branch of RNAi) definitely "learns" from an external agent (a virus) by generating siRNAs complementary to viral genes [40-42], a process that could be related, at least metaphorically, to Lamarck's "change of habits". Moreover, there is a degree of memory in the system because in many organisms siRNAs are amplified, and the resistance to the cognate virus can persist for several generations [43,44]. Such persistence of siRNA is one of the manifestations of increasingly recognized RNA-mediated inheritance, sometimes called paramutation [45,46]. The key difference from CASS is that (as far as currently known) siRNAs are not incorporated into the genome, so Lamarckian-type epigenetic inheritance but not bona fide genetic inheritance seems to be involved.

However, even that distinction becomes questionable in the case of transposon-derived piRNAs which form rapidly proliferating clusters that provide defense against transposable elements in the germ lines of all animals [47,48]. In the case of piRNA, like with the CRISPR-Cas, fragments of mobile element genomes are integrated into the host genome where they rapidly proliferate, apparently, under the pressure of selection for effective defense [48]. All the criteria for the IAC and the Lamarckian mode of evolution seem to be met by this system. It seems particularly remarkable that the sequestered germline, a crucial animal innovation, that seems to hamper some forms of Lamarckian inheritance, such as those associated with HGT, itself evolved a specific version of IAC.

Notably, recent findings in both plants and arthropods, although preliminary, indicate that these eukaryotes integrate virus-specific DNA into their genomes and might employ these integrated sequences to produce siRNAs that confer immunity to cognate viruses [49,50]. If corroborated by more detailed research, these mechanisms will be fully analogous to CRISPR-Cas and decidedly Lamarckian.

#### Horizontal gene transfer: a major Lamarckian component

Arguably, the most fundamental novelty brought about by comparative genomics in the last decade is the demonstration of the ubiquity and high frequency of horizontal gene transfer (HGT) among prokaryotes, and a considerable level of HGT in unicellular eukaryotes as well [51-56]. Prokaryotes readily obtain DNA from the environment, with phages and plasmids serving as vehicles, but in many cases, also directly, through the transformation pathway [57]. The absorbed DNA often integrates into prokaryotic chromosomes and can be fixed in a population if the transferred genetic material confers even a slight selective advantage onto the recipient, or even neutrally[58]. The HGT phenomenon has an obvious Lamarckian aspect to it: DNA is acquired from the environment, and naturally, the likelihood to acquire a gene that is abundant in the given habitat is much greater than the likelihood to receive a rare gene. The second component of the Lamarckian scheme, the direct adaptive value of the acquired character, is not manifest in all fixed HGT events but is relevant and common enough.

Perhaps, the most straightforward and familiar case in point is evolution of antibiotic resistance. When a sensitive prokaryote enters an environment where an antibiotic is present, the only chance for the newcomer to survive is to acquire a resistance gene(s) by HGT, typically, via a plasmid [59]. This common (and, of course, extremely practically important) phenomenon seems to be a clear case of Lamarckian inheritance. Indeed, a trait, in this case, the activity of the transferred gene that mediates antibiotic resistance, is acquired under a direct influence of the environment and is clearly advantageous, even essential in this particular niche.

More generally, any instance of HGT when the acquired gene provides an advantage to the recipient, in terms of reproduction in the given environment (that is specifically conducive to the transfer of the gene in question), seems to meet the Lamarckian criteria. Recent comparative-genomic studies indicate that HGT is the principal mode of bacterial adaptation to the environment through the extension of metabolic and signaling networks that integrate new, horizontally acquired genes and hence incorporate new capabilities within pre-existing frameworks [60-62]. Quantitatively, in prokaryotes, HGT appears to be a far more important route of adaptation than gene duplication [62,63].

A provocative indication that HGT might be an adaptive phenomenon is the recent discovery of the Gene Transfer Agents (GTAs). The GTAs are derivatives of defective bacteriophages that pack a variety of bacterial genes and transfer them within bacterial and archaeal populations [64,65]. The properties of GTAs remain to be investigated in detail but it seems to be a distinct possibility that these agents are dedicated vehicles of HGT that evolved under the selective pressure to enhance gene transfer. Should that be the case, one would have to conclude that HGT itself is, in part, an adaptive phenomenon.

### Stress-induced mutagenesis and activation of mobile elements: quasi-Lamarckian phenomena

Darwin emphasized the evolutionary importance of genuinely random, undirected variation whereas the Lamarckian modality of evolution is centered at directed variation that is specifically caused by environmental factors. The real evolution seems to defy such oppositions. A crucial case in point is the complex of diverse phenomena that collectively can be denoted stress-induced mutagenesis[66,67], one major facet of which is activation of mobile elements. In her classic experiments, McClintock demonstrated activation of "gene jumping" in plants under stress and the importance of this stress-induced mobility of distinct "controlling elements" for the emergence of resistance phenotypes [68,69].

The later, also famous and controversial, experiment of Cairns and coworkers on reversion of mutations in the *lac *operon induced by lactose brought the Lamarckian mechanism of evolution to the fore in a dramatic fashion [70,71]. Cairns et al. showed strong enhancement of frameshift reversion in the *lac *operon in the presence of lactose and boldly speculated that the classical Lamarckian mechanism of evolution was responsible for the observed effect, i.e., that lactose directly and specifically caused mutations in the lac operon. Subsequent, more thorough investigations, including the work of Cairns and Foster, showed that this was not the case: stress such as starvation was shown to induce mutations but not in specific loci [72-77]. Crucially, the mutations underlying the reversion of the *lac- *phenotype and other similar phenotypes have been shown to be strictly stress-induced: *lac- *cells plated on a medium with lactose as the only carbon source experience starvation stress - rather than emerging from the pool of pre-existing rare, spontaneous mutations [78-80].

Actually, stress-induced mutagenesis, specifically, the mutagenic SOS repair pathway in *E. coli *was discovered long before the experiments of Cairns. Moreover, Radman [81] and Echols [82] independently came up with the seminal idea that this mutagenic form of repair actually could be an adaptive, anti-stress response mechanism rather than malfunctioning of the repair systems. The two decades of subsequent research seem to prove this striking conjecture beyond reasonable doubt.

The adaptive character of error-prone DNA repair is supported by several lines of strong evidence. The activity of the SOS pathway and the other mutagenic repair mechanisms are elaborately regulated, in particular, through the switch from high-fidelity to error-prone double-strand break repair affected by the dedicated σ-factor, RpoS, apparently, to produce the optimal mutation rate [83]. Mutations produced by error-prone repair processes, although not targeted to specific genes, are not randomly scattered in the genome either. On the contrary, these mutations are clustered around double-stranded breaks, a phenomenon that is thought to have evolved as a distinct adaptation that allows coordinated evolvability of clustered, functionally linked genes (a central feature of genome architecture in prokaryotes) in rare cells where beneficial mutations emerge while limiting the damage to other parts of the genome [67,83-86]. More recently, stress-induced mutagenesis, in particular, retrotransposon mobilization, was demonstrated also in yeast and in animals [87-89], suggesting that this mechanism of adaptive evolvability is general across the entire range of cellular life forms [67].

Stress-induced mutagenesis is a rule among bacteria rather than an exception: among hundreds investigated natural isolates of *E. coli*, more than 80% showed induced mutagenesis in aged colonies, and the excess of stress-induced mutations over constitutive ones varied by several orders of magnitude [90].

Strikingly, it appears that stress-induced genome instability is also central to the progression of cancer in animals [82]. Tumors evolve under conditions of perpetual hypoxic stress which induces extensive genome rearrangement and mutation [91,92]. These stress-induced changes comprise the basis for the survival of mutants that are capable of uncontrolled growth in spite of the stress. Despite the differences in the actual mechanisms of mutagenic repair and its regulation, malignant tumors in animals are conceptually not so different from bacterial populations evolving under stress [67].

Adaptive evolution resulting from stress-induced mutagenesis is not exactly Lamarckian because the stress does not cause mutations directly and specifically in genes conferring stress resistance. Instead, organisms evolved mechanisms that in response to stress induce non-specific mutagenesis which, however, appears to be fine-tuned in such a way so to minimize the damage from deleterious mutations in those rare genomes that carry a beneficial mutation. This type of mechanism is best defined as quasi-lamarckian. Indeed, in the case of stress-induced mutagenesis: i) mutations are triggered by environmental conditions; ii) the induced mutations lead to adaptation to the stress factor(s) that triggered mutagenesis; iii) mutagenic repair is subject to elaborate regulation which leaves no reasonable doubt regarding the adaptive nature of this process.

Remarkably, there is a direct link between the Lamarckian aspects of stress-induced mutagenesis and HGT via the phenomenon of antibiotic-induced HGT of resistance determinants [93,94]. More specifically, many antibiotics induce the SOS response which in turn leads to the mobilization of integrating conjugative elements (ICEs) that serve as vehicles for the antibiotic resistance genes. Here we observe an apparent convergence of different mechanisms of the genome change in the Lamarckian modality.

### The dissolution of a conflict: the continuum of Darwinian and Lamarckian mechanisms of evolution

In the preceding sections, we discussed a considerable variety of phenomena some of which seem to strictly meet the Lamarckian criteria whereas others qualify in quasi-Lamarckian (Table 1). The crucial difference between "Darwinian" and "Lamarckian" mechanisms of evolution is that the former emphasizes random, undirected variation whereas the latter is based on variation directly caused by an environmental cue and resulting in a specific response to that cue (Figure 1). Neither Lamarck nor Darwin were aware of the mechanisms of emergence and fixation of heritable variation. Therefore, it was relatively easy for Lamarck to entertain the idea that phenotypic variation directly translates into heritable (what we now consider genetic or genomic) changes. We now realize that the strict Lamarckian scenario is extremely demanding in that a molecular mechanism must exist for the effect of a phenotypic change to be channeled into the corresponding modification of the genome (mutation). There seems to be no general mechanisms for such reverse genome engineering and it is not unreasonable to surmise that genomes are actually protected from this type of mutation. The "central dogma of molecular biology" which states that there is no information flow from protein to nucleic acids [95] is a partial embodiment of this situation. However, in principle, the backward flow of specific information from the phenotype - or the environment viewed as extended phenotype - to the genome is not impossible owing to the wide spread of reverse transcription and DNA transposition. Highly sophisticated mechanisms are required for this bona fide Lamarckian scenario to work, and in two remarkable cases, the CASS and the piRNA system, such mechanisms have been discovered.

**Table 1:** Lamarckian and quasi-Lamarckian phenomena

Phenomenon	Biological role/function	Phyletic spread	Lamarckian criteria		
			**Genomic changes caused by environmental factor**	**Changes are specific to relevant genomic loci**	**Changes provide adaptation to the causative factor**

Bona fide Lamarckian					

CRISPR-Cas	Defense against viruses and other mobile elements	Archaea and bacteria (present in ~1/3 sequenced genomes)	Yes	Yes	Yes

piRNA	Defense against transposable elements in germline	Animals (apparently, all)	Yes	Yes	Yes

HGT (specific cases)	Adaptation to new environment, stress response, resistance	Archaea, bacteria, unicellular eukaryotes	Yes	Yes	Yes

Quasi-Lamarckian					

HGT (general phenomenon)	Diverse innovations	Archaea, bacteria, unicellular eukaryotes	Yes	No	Yes/no

Stress-induced mutagenesis	Stress response/resistance/ adaptation to new conditions	Ubiquitous	Yes	No or partially	Yes (but general evolvability/ Adaptability enhanced as well)

Although the existence of other bona fide Lamarckian systems, beyond the CASS and the piRNA, is imaginable and even likely, as suggested, for instance, by the discovery of virus-specific sequences, potentially conferring resistance to the cognate viruses, in plant and animal genomes [49,50] these mechanisms hardly constitute the mainstream of genome evolution. In contrast, the mechanisms that we denoted in the preceding sections as quasi-Lamarckian are ubiquitous. Conceptually, these mechanisms seem to be no less remarkable - and no less sophisticated - than the genuine Lamarckian scenario, because the quasi-Lamarckian processes translate mutations that, in and by themselves, are random into specific, adaptive responses to environmental cues.

**Figure 3:**
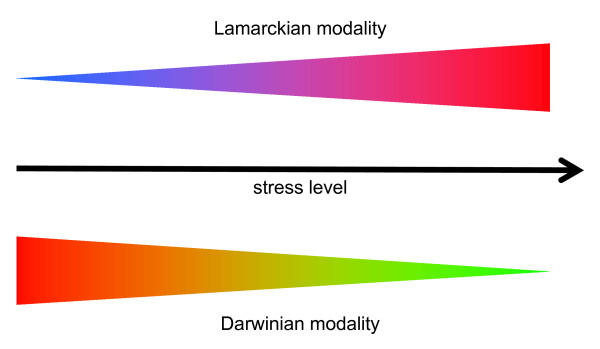
**Environment, stress and the Lamarckian and Darwinian modalities of evolution**.

The theme of powerful, often adverse effects of the environment on organisms seems to be common to different facets of the Lamarckian mode of evolution described here, be it the case of the CASS system or stress induced mutagenesis. This association is most likely not spurious: it stands to reason that strong signals from the environment trigger (quasi)Lamarckian processes whereas relatively weak signals ("business as usual") are conducive to the Darwinian modality of evolution (Figure 3).

In a recent discussion of the evolutionary significance of HGT [96], Poole suggested that the Lamarckian aspect of HGT, which was invoked by Goldenfeld and Woese [56]as the dominant modality of the earliest stages of life evolution, becomes illusory when "a gene's view" of evolution [97] is adopted. Indeed, it appears that the Lamarckian modality is associated primarily, if not exclusively, with the organismal level of complexity, and does not apply to the most fundamental level of evolution which indeed involves genes, independently evolving portions of genes (e.g. those encoding distinct protein domains) and mobile elements [98]. In that sense, Lamarckian evolution may be considered an "emergent phenomenon", perhaps, not surprisingly, considering the need for complex mechanisms for the integration of new material into the genome, to realize the Lamarckian scheme.

In our opinion, the view of directed and undirected variation and their places in evolution presented here diffuses the long-standing tension between the Darwinian and Lamarckian scenarios. Indeed, evolution is a continuum of processes, from genuinely random to those that are exquisitely orchestrated to ensure a specific response to a particular challenge. The critical realization suggested by many recent advances referred to in this article is that genomic variation is a far more complex phenomenon than previously imagined and is regulated at multiple levels to provide adaptive reactions to changes in the environment. The distinction between Lamarckian and Darwinian mechanisms of evolution potentially could be considered as one of only historical, semantic or philosophical interest. However, the radical reappraisal of the nature of genomic variation and the realization that much of this variation is adaptive, thus apparently eliminating the conflict between the Lamarckian and Darwinian scenarios, is a veritable, although underappreciated paradigm shift in modern biology.

## Conclusion

A close examination of a variety of widespread processes that contribute to the generation of genomic variation shows that evolution does not rely entirely on stochastic mutation. Instead, generation of variation is often controlled via elaborate molecular machinery that instigates adaptive responses to environmental challenges of various degrees of specificity. Thus, genome evolution appears to span the entire spectrum of scenarios, from the purely Darwinian, based on random variation, to *bona fide *Lamarckian where a specific mechanism of response to a cue is fixed in an evolving population through a distinct modification of the genome. In a broad sense, all these routes of genomic variation reflect the interaction between the evolving population and the environment in which the active role belongs either to selection alone (pure Darwinian scenario) or to directed variation that itself may become the target of selection (Lamarckian scenario).

## Competing interests

The authors declare that they have no competing interests.

## Authors' contributions

EVK conceived of the article and wrote the original draft; YIW modified the manuscript and designed and prepared the figures; both authors read, edited and approved the final text.

## Reviewers' reports

### Reviewer 1: Juergen Brosius, University of Muenster

This is a timely, captivating and clear presentation of yet another and highly significant testimony to the fact that in nature, we rarely encounter clear boundaries. Figure 1 is a centerpiece of the article, as it clearly pinpoints the salient differences between Lamarckian, Darwinian and also neutral evolution, but at the same time it illustrates their great similarities. Key in the Lamarckian mode are the mutation-directing mechanisms. Although acquired traits can be passed onto the next generation in case of a greatly reduced Weismann barrier as would have been the case in an RNA world, where genotype and phenotype were almost indistinguishable on the same ribonucleic acid molecule [99], the directional component was almost certainly absent.

While commenting on Kammerer in the Background section, the authors might include that very recently A. Vargas has revisited Paul Kammerer's controversial midwife toad experiments. He comes to the conclusion that there might be substance to Kammerer's observations based upon what we learned about patterns of epigenetic inheritance, in the meantime [12]; see also commentaries by Wagner and Pennisi [13,14].

Authors' response: *We modified the text accordingly and cited these publications; the pointer to this recent re-analysis of Kammerer's work is greatly appreciated*.

It is also worth noting that memes [97] and cultural evolution in general obey the laws of both Darwinian *and *Lamarckian evolution [100]. Recently, it was proposed that the human lineage is at the verge of several major evolutionary transitions [101], one of these being a capability very close to Lamarckism with the potential to direct acquired knowledge on phenotype/genotype relationships into our germ-line, the tools of Genetic Engineering and Molecular Medicine representing *the *mutation directing mechanisms [99,102]. Hence, I would recommend to qualify the sentence on page 20: "There seems to be no general mechanisms for such reverse genome engineering and it is not unreasonable to surmise that genomes are actually protected from this type of mutation" with "up to now".

Authors' response: *These are interesting possibilities but we are of the opinion that, when and if realized, these aritficial methods of introducing directed changes into genomes will be qualitatively distinct from naturally evolved mechanisms. Accordingly, we did not modify the text of the article in the belief that the reader is adequately served by this comment*.

However, we do not need to wait for this to fully develop. The authors recognized that a mechanism of capturing invader nucleic acids (e.g., from viruses and plasmids) and using it antisense to the genetic material that evolved in Archaea and Bacteria long ago is very close to the definition of Lamarckism. This mechanism is well described in the article. Although there are a number of original papers and reviews on the subject, no one seems to have recognized the significance of the findings with respect to Lamarckism (but see ref. 24 and Acknowledgements).

Concerning definitions regarding "adaptation to the original causative factor" or the "adaptive reaction", at least initially, this is not always the case: Strictly speaking, the CRISPR system is an exaptation. For example, the viral sequences did not evolve for the function in the host; instead the host is co-opting them subsequent to integration for RNA-based antivirus immunity. Perhaps one way out would be the use of the term "aptation" which comprises exaptation and adaptation as suggested by Gould and Vrba [103].

Authors' response: *We think this is a very subtle although, perhaps, valid semantic point. Again, the interested reader will be alerted by the comment*.

Horizontal gene transfer (HGT), which was rampant in the RNA world [99], I would not hang up too high with respect to Lamarckism. The CRISPR system is a much more impressive example. With respect to HGT, once more I only see a continuum with HGT on one end and sex among members of the same species on the other. HGT is just limit-, border-, or barrierless sex acquiring different genes instead of different alleles [99].

Obviously, I do not quite agree with the view that the "Lamarckian modality is associated primarily, if not exclusively, with the organismal level of complexity, and does not apply to the most fundamental level of evolution which indeed involves genes, independently evolving portions of genes (e.g. those encoding distinct protein domains) and mobile elements [98]" because of the inseparability of genotype and phenotype in the RNA world [99]. However, I agree with the authors to consider Lamarckism as largely an "emergent phenomenon" (but see the CRISPR system) in our lineage (see memes and other evolutionary transitions discussed above).

Stress-induced mutations, whether point mutations including small indels including SOS repair or large indels in the form of mobile genetic elements constitute a crude machinery, at best, but hardly directed. Despite a preference for TTAAAA during RNA mediated retroposition in placental mammals [104], insertions can happen at almost any locus and hardly can be considered specific. At a later point, the authors put this in the right perspective. I hope misguided individuals do not stop reading before they reach these important paragraphs. Giving an outlook on the future of our species, we might expect a sharp increase in mutations and retroposition, due to the self-inflicted stress by feedback from our environment.

Once more, one can only agree with Stephen Jay Gould: ". our deepest puzzles and most fascinating inquiries often fall into a no-man's land not clearly commanded by either party" [7].

### Reviewer 2: Valerian Dolja, Oregon State University

I follow the recent series of Eugene Koonin's conceptual papers pretty closely, and I must admit that this latest one is a surprising twist. When we were taught Biology, work of Lamarck appeared to be a fine example of a feasible, coherent, and even likable theory that, however, had no experimental support whatsoever. By and large, this perception did not change in the last four decades of our direct engagement in biology research.

Enter discovery of the CRISPR system based initially on the bioinformatics analysis of the prokaryotic genomes by Koonin's team, and then confirmed experimentally in several labs, again, with Koonin's direct involvement. Even though in its infancy (e.g., it is not known how phages respond to this defense; they either have CRISPR suppressors or are busy evolving those), CRISPR already emerged as a truly Lamarckian phenomenon, complete with a mechanism for insertion of the acquired phage DNA fragments into bacterial genomes. With the addition of piRNA facet of RNAi system and other, 'quasi-Lamarckian' phenomena such as HGT (particularly when mediated by GTAs), inheritance of the environmental DNA becomes a major player in, at least, evolution of prokaryotes.

However, one can still ask how relevant this partial vindication of Lamarck is to the contemporary, mechanism-based understanding of biological evolution. One argument is that, vili-nili, Lamarck and Darwin based their concepts solely on observational natural philosophy rather than on investigation of underlying molecular mechanisms. It seems that the latter beats the former; suffice it to say that the Mendel laws are trivial consequence of the DNA replication mechanisms. In a sense, it does not matter so much, Darwinian or Lamarckian, when it is understood how evolution operates at the molecular, organismal, and population levels.

This having been said, I still believe that the effort of reviving Lamarck's ideas should be applauded for at least the following reasons. Firstly, it enriches the conceptual framework of modern evolutionary theory by providing a novel insight into complexity of relationships between genomes and environment, and by showing several amazing examples of how the latter can directly or indirectly change the former. It is also fitting that Koonin who co-fathered discovery of the CRISPR system, has also brought this, now molecular mechanism-based Lamarckism back to the fold of evolutionary biology. Secondly, it shows how even the seemingly opposing theories can be combined to complement each other. This pluralistic approach appears to be a strong and continuing trend in Koonin's work, be it introns early vs late or TOL vs FOL debates. Thirdly, it emphasizes the need and the benefits of continuous rethinking and reinterpreting the history of science. The significance of the latter issue is hard to overestimate given the dramatic personal story of Kammerer (recently recapitulated in *Science*) that intertwined with the darkest days of Russian biology under Stalin and Lysenko.

In conclusion, I think that Koonin and Wolf essay will be very instructive for the broad audience of the students of evolution and their opponents alike. It integrates so seamlessly the literary, historical, philosophical, and mechanistic approaches. It also helps a lot that the paper is very engaging, impossible to put aside before finishing.

Authors' response: *We appreciate the constructive comments and would like to emphasize that the primary goal of this paper is indeed not a reappraisal of the role of Jean-Bapteste Lamarck in the history of evolutionary biology. To engage in such an undertaking, one needs to be a professional historian of science, which we certainly are not, and of course, to be able to read Lamarck's oeuvre in the original which, most unfortunately, we cannot do (at least, not without a long-term, sustained effort). Rather, this paper focuses on the increasing realization of the more direct and active involvement of environmental factors in evolutionarily relevant genomic change than perceived within the Modern Synthesis of Evolutionary Biology. This emerging new aspect of evolution necessarily brings to mind Lamarck but we do not propound a revival of the actual ideas of Philosophie Zoologique*.

### Reviewer 3: Martijn Huynen, Radboud University

Koonin and Wolf have written an interesting and provocative study on the Lamarckian aspects of some non-random genetic changes. In commenting on this paper I will try to not run into semantic issues about what is really Lamarckian.

Some newly discovered systems like the CAS system can, also in my view, clearly be regarded as Lamarckian, and I applaud the authors for carefully making their case. To regard Horizontal Gene Transfer (HGT) as Lamarckian one would however have to show that a substantial fraction of HGT is indeed adaptive. I do not think we have data to substantiate that. One could of course argue that species living in the same environment share the same needs, like adaptation to high temperatures, and thus the transfer of Reverse Gyrase from Archaea to Bacteria could be regarded as Lamarckian. I doubt however that of the total number of genes that get transferred a reasonable fraction will have adaptive value. It may be tentative to think so, but we simply have no data to separate the effects of the process of HGT from the process + the effect of selection.

I would therefore not agree that "any instance of HGT when the acquired gene provides an advantage to the recipient, in terms of reproduction in the given environment (that is specifically conducive to the transfer of the gene in question), seems to meet the Lamarckian criteria", because there will be many non-adaptive HGTs, just as there are many non-adaptive mutations.

Authors' response: *we do not claim that all or most of HGT is adaptive or Lamarckian but only that there is a substantial Lamarckian component to it. The quoted sentence says nothing about the frequency of adaptive HGT, so we maintain that it is valid. Further, one has to clearly distinguish between the occurrence of HGT and its fixation in the population. Of course, the huge majority of occurring HGT is non-adaptive but that does not necessarily apply to the fixed transfers*.

Similarly I do not think that there is evidence to support that the stress induced changes in tumors are adaptive in themselves, even though some of them could indeed be selected, and I do not know of any evidence to support that "the induced mutations lead to adaptation to the stress factor(s) that triggered mutagenesis".

Authors' response: *it is important to emphasize that, unlike the case of CRISPR and the adaptive component of HGT, which we view as bona fide Lamarckian, we denote stress-induced mutagenesis including that occurring in tumors, a quasi-Lamarckian phenomenon *(Table 1). *So we do not posit that induced mutations are adaptive "in themselves" but rather that some of them are, often, only a small fraction. However, all these mutations are directly induced by environmental stress factors, and those that are adaptive, even if a small minority, are most consequential for evolution*.

Finally: at least I do not realize that "much of this variation is adaptive". But this study did get me to think about it, and as such I think this manuscript provides valuable new insights and thoughts about the possible continuum between Darwinian and Lamarckian evolution.

## References

[B1] Darwin C On the Origin of Species 1859 1 London: Murray

[B2] Lamarck J-B Philosophie zoologique, ou exposition des considérations relatives à l'histoire naturelle des animaux 1809 Paris: Dentu

[B3] Graur D Gouy M Wool D In retrospect: Lamarck's treatise at 200 Nature 2009 460 7256 688 689

[B4] Darwin C Origin of Species 1872 6 New York: The Modern Library

[B5] Packard AS Lamarck, the founder of evolution: His life and work 1901 New York: Longmans, Green & Co

[B6] Burkhard RW Spirit of System: Lamarck and Evolutionary Biology 1995 Cambridge, MA: Harvard Univ Press

[B7] Gould SJ The Structure of Evolutionary Theory 2002 Cambrdige, MA: Harvard Univ. Press

[B8] Bulmer M Did Jenkin's swamping argument invalidate Darwin's theory of natural selection? British Journal of History of Science 2004 37 281 297

[B9] Weismann A Essays Upon Heredity 1889 Oxford: Clarendon Press

[B10] Aronson LR The case of The Case of the Midwife Toad Behav Genet 1975 5 2 115 125 1093540 10.1007/BF01066805

[B11] Gliboff S "Protoplasm...is soft wax in our hands": Paul Kammerer and the art of biological transformation Endeavour 2005 29 4 162 167 16271762 10.1016/j.endeavour.2005.10.001

[B12] Vargas AO Did Paul Kammerer discover epigenetic inheritance? A modern look at the controversial midwife toad experiments J Exp Zool B Mol Dev Evol 2009 312 7 667 678 19731234 10.1002/jez.b.21319

[B13] Wagner GP Paul Kammerer's midwife toads: about the reliability of experiments and our ability to make sense of them J Exp Zool B Mol Dev Evol 2009 312 7 665 666 19790195 10.1002/jez.b.21324

[B14] Pennisi E History of science. The case of the midwife toad: fraud or epigenetics? Science 2009 325 5945 1194 1195 19729631 10.1126/science.325_1194

[B15] Medvedev ZA The rise and fall of T.D. Lysenko 1969 New York: Columbia University Press

[B16] Soyfer VN The consequences of political dictatorship for Russian science Nat Rev Genet 2001 2 9 723 729 11533721 10.1038/35088598

[B17] Soyfer VN Lysenko and the tragedy of Soveit science 1994 New Brunswick, NJ: Rutgers Univ Press

[B18] O Polozhenii v Biologicheskoi Nauke. Stenograficheskii otchet sessii Vsesoyuznoi Akademii Selskohozyastvennyh Nauk imeni V. I. Lenina (On the Situation in Biological Science. A transcript of the Session of the V. I. Lenin All-Union Academy of Agricultural Sciences, July 31 - August 7, 1948) 1948 Moscow, USSR: The State Agricultural Literature Publishers

[B19] Gould sj Full House: The Spread of excellence from Plato to Darwin 1997 New York: Three Rivers Press

[B20] Lynch M The frailty of adaptive hypotheses for the origins of organismal complexity Proc Natl Acad Sci USA 2007 104 Suppl 1 8597 8604 17494740 10.1073/pnas.0702207104 PMC1876435

[B21] Lynch M The origins of genome archiecture 2007 Sunderland, MA: Sinauer Associates

[B22] Koonin EV Darwinian evolution in the light of genomics Nucleic Acids Res 2009 37 2 1011 1034 19213802 10.1093/nar/gkp089 PMC2651812

[B23] Wright S Evolution: Selected papers 1986 Chicago: Univ Chicago Press

[B24] Sorek R Kunin V Hugenholtz P CRISPR--a widespread system that provides acquired resistance against phages in bacteria and archaea Nat Rev Microbiol 2008 6 3 181 186 18157154 10.1038/nrmicro1793

[B25] Waters LS Storz G Regulatory RNAs in bacteria Cell 2009 136 4 615 628 19239884 10.1016/j.cell.2009.01.043 PMC3132550

[B26] Oost J van der Jore MM Westra ER Lundgren M Brouns SJ CRISPR-based adaptive and heritable immunity in prokaryotes Trends Biochem Sci 2009 34 8 401 407 19646880 10.1016/j.tibs.2009.05.002

[B27] Jansen R Embden JD Gaastra W Schouls LM Identification of genes that are associated with DNA repeats in prokaryotes Mol Microbiol 2002 43 6 1565 1575 11952905 10.1046/j.1365-2958.2002.02839.x

[B28] Makarova KS Aravind L Grishin NV Rogozin IB Koonin EV A DNA repair system specific for thermophilic archaea and bacteria predicted by genomic context analysis Nucleic Acids Res 2002 30 482 496 11788711 10.1093/nar/30.2.482 PMC99818

[B29] Haft DH Selengut J Mongodin EF Nelson KE A guild of 45 CRISPR-associated (Cas) protein families and multiple CRISPR/Cas subtypes exist in prokaryotic genomes PLoS Comput Biol 2005 1 6 e60 16292354 10.1371/journal.pcbi.0010060 PMC1282333

[B30] Mojica FJ Diez-Villasenor C Garcia-Martinez J Soria E Intervening sequences of regularly spaced prokaryotic repeats derive from foreign genetic elements J Mol Evol 2005 60 2 174 182 15791728 10.1007/s00239-004-0046-3

[B31] Pourcel C Salvignol G Vergnaud G CRISPR elements in Yersinia pestis acquire new repeats by preferential uptake of bacteriophage DNA, and provide additional tools for evolutionary studies Microbiology 2005 151 Pt 3 653 663 15758212 10.1099/mic.0.27437-0

[B32] Makarova KS Grishin NV Shabalina SA Wolf YI Koonin EV A putative RNA-interference-based immune system in prokaryotes: computational analysis of the predicted enzymatic machinery, functional analogies with eukaryotic RNAi, and hypothetical mechanisms of action Biol Direct 2006 1 7 16545108 10.1186/1745-6150-1-7 PMC1462988

[B33] Barrangou R Fremaux C Deveau H Richards M Boyaval P Moineau S Romero DA Horvath P CRISPR provides acquired resistance against viruses in prokaryotes Science 2007 315 5819 1709 1712 17379808 10.1126/science.1138140

[B34] Brouns SJ Jore MM Lundgren M Westra ER Slijkhuis RJ Snijders AP Dickman MJ Makarova KS Koonin EV Oost J van der Small CRISPR RNAs guide antiviral defense in prokaryotes Science 2008 321 5891 960 964 18703739 10.1126/science.1159689 PMC5898235

[B35] Hale C Kleppe K Terns RM Terns MP Prokaryotic silencing (psi)RNAs in *Pyrococcus furiosus* Rna 2008 14 12 2572 2579 18971321 10.1261/rna.1246808 PMC2590957

[B36] Wiedenheft B Zhou K Jinek M Coyle SM Ma W Doudna JA Structural basis for DNase activity of a conserved protein implicated in CRISPR-mediated genome defense Structure 2009 17 6 904 912 19523907 10.1016/j.str.2009.03.019

[B37] Andersson AF Banfield JF Virus population dynamics and acquired virus resistance in natural microbial communities Science 2008 320 5879 1047 1050 18497291 10.1126/science.1157358

[B38] Tyson GW Banfield JF Rapidly evolving CRISPRs implicated in acquired resistance of microorganisms to viruses Environ Microbiol 2008 10 1 200 207 17894817 10.1111/j.1462-2920.2007.01444.x

[B39] Marraffini LA Sontheimer EJ CRISPR interference limits horizontal gene transfer in staphylococci by targeting DNA Science 2008 322 5909 1843 1845 19095942 10.1126/science.1165771 PMC2695655

[B40] van Rij RP Berezikov E Small RNAs and the control of transposons and viruses in Drosophila Trends Microbiol 2009 17 4 163 171 19299135 10.1016/j.tim.2009.01.003

[B41] Carthew RW Sontheimer EJ Origins and Mechanisms of miRNAs and siRNAs Cell 2009 136 4 642 655 19239886 10.1016/j.cell.2009.01.035 PMC2675692

[B42] Voinnet O Origin, biogenesis, and activity of plant microRNAs Cell 2009 136 4 669 687 19239888 10.1016/j.cell.2009.01.046

[B43] Grishok A Tabara H Mello CC Genetic requirements for inheritance of RNAi in *C. elegans* Science 2000 287 5462 2494 2497 10741970 10.1126/science.287.5462.2494

[B44] Alcazar RM Lin R Fire AZ Transmission dynamics of heritable silencing induced by double-stranded RNA in *Caenorhabditis elegans* Genetics 2008 180 3 1275 1288 18757930 10.1534/genetics.108.089433 PMC2581934

[B45] Chandler VL Paramutation: from maize to mice Cell 2007 128 4 641 645 17320501 10.1016/j.cell.2007.02.007

[B46] Hollick J Paramutation and Development Annu Rev Cell Dev Biol 2009 19575656 10.1146/annurev.cellbio.042308.113400

[B47] Aravin AA Hannon GJ Brennecke J The Piwi-piRNA pathway provides an adaptive defense in the transposon arms race Science 2007 318 5851 761 764 17975059 10.1126/science.1146484

[B48] Assis R Kondrashov AS Rapid repetitive element-mediated expansion of piRNA clusters in mammalian evolution Proc Natl Acad Sci USA 2009 106 17 7079 7082 19357307 10.1073/pnas.0900523106 PMC2667148

[B49] Bertsch C Beuve M Dolja VV Wirth M Pelsy F Herrbach E Lemaire O Retention of the virus-derived sequences in the nuclear genome of grapevine as a potential pathway to virus resistance Biol Direct 2009 4 21 19558678 10.1186/1745-6150-4-21 PMC2714080

[B50] Flegel TW Hypothesis for heritable, anti-viral immunity in crustaceans and insects Biol Direct 2009 4 32 19725947 10.1186/1745-6150-4-32 PMC2757015

[B51] Doolittle WF Lateral genomics Trends Cell Biol 1999 9 12 M5 8 10611671

[B52] Doolittle WF Zhaxybayeva O On the origin of prokaryotic species Genome Res 2009 19 5 744 756 19411599 10.1101/gr.086645.108

[B53] Koonin EV Makarova KS Aravind L Horizontal gene transfer in prokaryotes: quantification and classification Annu Rev Microbiol 2001 55 709 742 11544372 10.1146/annurev.micro.55.1.709 PMC4781227

[B54] Gogarten JP Townsend JP Horizontal gene transfer, genome innovation and evolution Nat Rev Microbiol 2005 3 9 679 687 16138096 10.1038/nrmicro1204

[B55] Koonin EV Wolf YI Genomics of bacteria and archaea: the emerging dynamic view of the prokaryotic world Nucleic Acids Res 2008 36 21 6688 6719 18948295 10.1093/nar/gkn668 PMC2588523

[B56] Goldenfeld N Woese C Biology's next revolution Nature 2007 445 7126 369 17251963 10.1038/445369a

[B57] Bushman F Lateral DNA Transfer: Mechanisms and Consequences 2001 Cold Spring Harbor, NY: Cold Spring Harbor Laboratory Press

[B58] Novozhilov AS Karev GP Koonin EV Mathematical modeling of evolution of horizontally transferred genes Mol Biol Evol 2005 22 8 1721 1732 15901840 10.1093/molbev/msi167

[B59] Barlow M What antimicrobial resistance has taught us about horizontal gene transfer Methods Mol Biol 2009 532 397 411 19271198 10.1007/978-1-60327-853-9_23

[B60] Homma K Fukuchi S Nakamura Y Gojobori T Nishikawa K Gene cluster analysis method identifies horizontally transferred genes with high reliability and indicates that they provide the main mechanism of operon gain in 8 species of gamma-Proteobacteria Mol Biol Evol 2007 24 3 805 813 17185745 10.1093/molbev/msl206

[B61] Davids W Zhang Z The impact of horizontal gene transfer in shaping operons and protein interaction networks--direct evidence of preferential attachment BMC Evol Biol 2008 8 23 18218112 10.1186/1471-2148-8-23 PMC2259305

[B62] Maslov S Krishna S Pang TY Sneppen K Toolbox model of evolution of prokaryotic metabolic networks and their regulation Proc Natl Acad Sci USA 2009 106 24 9743 9748 19482938 10.1073/pnas.0903206106 PMC2701025

[B63] Pal C Papp B Lercher MJ Adaptive evolution of bacterial metabolic networks by horizontal gene transfer Nat Genet 2005 37 12 1372 1375 16311593 10.1038/ng1686

[B64] Lang AS Beatty JT Importance of widespread gene transfer agent genes in alpha-proteobacteria Trends Microbiol 2007 15 2 54 62 17184993 10.1016/j.tim.2006.12.001

[B65] Stanton TB Prophage-like gene transfer agents-novel mechanisms of gene exchange for Methanococcus, Desulfovibrio, Brachyspira, and Rhodobacter species Anaerobe 2007 13 2 43 49 17513139 10.1016/j.anaerobe.2007.03.004

[B66] Foster PL Stress-induced mutagenesis in bacteria Crit Rev Biochem Mol Biol 2007 42 5 373 397 17917873 10.1080/10409230701648494 PMC2747772

[B67] Galhardo RS Hastings PJ Rosenberg SM Mutation as a stress response and the regulation of evolvability Crit Rev Biochem Mol Biol 2007 42 5 399 435 17917874 10.1080/10409230701648502 PMC3319127

[B68] McClintock B The origin and behavior of mutable loci in maize Proc Natl Acad Sci USA 1950 36 6 344 355 15430309 10.1073/pnas.36.6.344 PMC1063197

[B69] McClintock B The significance of responses of the genome to challenge Science 1984 226 4676 792 801 15739260 10.1126/science.15739260

[B70] Cairns J Overbaugh J Miller S The origin of mutants Nature 1988 335 6186 142 145 3045565 10.1038/335142a0

[B71] Stahl FW Genetics. If it smells like a unicorn Nature 1990 346 6287 791 2202904 10.1038/346791a0

[B72] Foster PL Cairns J Mechanisms of directed mutation Genetics 1992 131 4 783 789 1516815 10.1093/genetics/131.4.783 PMC1205091

[B73] Cairns J Directed mutation Science 1993 260 5112 1221 1224 8493560 10.1126/science.8493560

[B74] Torkelson J Harris RS Lombardo MJ Nagendran J Thulin C Rosenberg SM Genome-wide hypermutation in a subpopulation of stationary-phase cells underlies recombination-dependent adaptive mutation Embo J 1997 16 11 3303 3311 9214645 10.1093/emboj/16.11.3303 PMC1169946

[B75] Lombardo MJ Torkelson J Bull HJ McKenzie GJ Rosenberg SM Mechanisms of genome-wide hypermutation in stationary phase Ann N Y Acad Sci 1999 870 275 289 10415490 10.1111/j.1749-6632.1999.tb08888.x

[B76] Foster PL Adaptive mutation: has the unicorn landed? Genetics 1998 148 4 1453 1459 9560365 10.1093/genetics/148.4.1453 PMC1460081

[B77] Rosenberg SM Mutation for survival Curr Opin Genet Dev 1997 7 6 829 834 9468794 10.1016/s0959-437x(97)80047-0

[B78] Cairns J Foster PL Adaptive reversion of a frameshift mutation in Escherichia coli Genetics 1991 128 4 695 701 1916241 10.1093/genetics/128.4.695 PMC1204544

[B79] McKenzie GJ Lombardo MJ Rosenberg SM Recombination-dependent mutation in Escherichia coli occurs in stationary phase Genetics 1998 149 2 1163 1165 9735004 10.1093/genetics/149.2.1163 PMC1460184

[B80] Hastings PJ Bull HJ Klump JR Rosenberg SM Adaptive amplification: an inducible chromosomal instability mechanism Cell 2000 103 5 723 731 11114329 10.1016/s0092-8674(00)00176-8

[B81] Radman M Prakash L, Miller FSM, Lawrence C, Tabor HW Phenomenology of an inducible mutagenic DNA repair pathway in Escherichia coli: SOS repair hypothesis Molecular and Environmental aspects of Mutagenesis 1974 Springfield, Ill: Charles C. Thomas 128 142

[B82] Echols H SOS functions, cancer and inducible evolution Cell 1981 25 1 1 2 7023691 10.1016/0092-8674(81)90223-3

[B83] Ponder RG Fonville NC Rosenberg SM A switch from high-fidelity to error-prone DNA double-strand break repair underlies stress-induced mutation Mol Cell 2005 19 6 791 804 16168374 10.1016/j.molcel.2005.07.025

[B84] Bull HJ Lombardo MJ Rosenberg SM Stationary-phase mutation in the bacterial chromosome: recombination protein and DNA polymerase IV dependence Proc Natl Acad Sci USA 2001 98 15 8334 8341 11459972 10.1073/pnas.151009798 PMC37440

[B85] Wang J Gonzalez KD Scaringe WA Tsai K Liu N Gu D Li W Hill KA Sommer SS Evidence for mutation showers Proc Natl Acad Sci USA 2007 104 20 8403 8408 17485671 10.1073/pnas.0610902104 PMC1895962

[B86] Drake JW Too many mutants with multiple mutations Crit Rev Biochem Mol Biol 2007 42 4 247 258 17687667 10.1080/10409230701495631 PMC2265383

[B87] Datta A Jinks-Robertson S Association of increased spontaneous mutation rates with high levels of transcription in yeast Science 1995 268 5217 1616 1619 7777859 10.1126/science.7777859

[B88] Scholes DT Kenny AE Gamache ER Mou Z Curcio MJ Activation of a LTR-retrotransposon by telomere erosion Proc Natl Acad Sci USA 2003 100 26 15736 15741 14673098 10.1073/pnas.2136609100 PMC307637

[B89] Bindra RS Glazer PM Genetic instability and the tumor microenvironment: towards the concept of microenvironment-induced mutagenesis Mutat Res 2005 569 1-2 75 85 15603753 10.1016/j.mrfmmm.2004.03.013

[B90] Bjedov I Tenaillon O Gerard B Souza V Denamur E Radman M Taddei F Matic I Stress-induced mutagenesis in bacteria Science 2003 300 5624 1404 1409 12775833 10.1126/science.1082240

[B91] Glazer PM Bindra RS Introduction: the evolving picture of the hypoxic tumour microenvironment Curr Mol Med 2009 9 4 399 400 19519396 10.2174/156652409788167069

[B92] Huang LE Bindra RS Glazer PM Harris AL Hypoxia-induced genetic instability--a calculated mechanism underlying tumor progression J Mol Med 2007 85 2 139 148 17180667 10.1007/s00109-006-0133-6

[B93] Beaber JW Hochhut B Waldor MK SOS response promotes horizontal dissemination of antibiotic resistance genes Nature 2004 427 6969 72 74 14688795 10.1038/nature02241

[B94] Hastings PJ Rosenberg SM Slack A Antibiotic-induced lateral transfer of antibiotic resistance Trends Microbiol 2004 12 9 401 404 15337159 10.1016/j.tim.2004.07.003

[B95] Crick F Central dogma of molecular biology Nature 1970 227 5258 561 563 4913914 10.1038/227561a0

[B96] Poole AM Horizontal gene transfer and the earliest stages of the evolution of life Res Microbiol 2009 19647074 10.1016/j.resmic.2009.07.009

[B97] Dawkins R The Selfish Gene 1976 Oxford: Oxford University Press

[B98] Koonin EV Wolf YI The fundamental units, processes and patterns of evolution, and the Tree of Life conundrum Biology Direct 2009 4 33 19788730 10.1186/1745-6150-4-33 PMC2761301

[B99] Brosius J Gene duplication and other evolutionary strategies: from the RNA world to the future J Struct Funct Genomics 2003 3 1-4 1 17 12836680 10.1023/a:1022627311114

[B100] Gould SJ The panda's thumb 1980 New York: Norton

[B101] Brosius J Disparity, adaptation, exaptation, bookkeeping, and contingency at the genome level Paleobiology 2005 31 1 16

[B102] Brosius J From Eden to a hell of uniformity? Directed evolution in humans Bioessays 2003 25 8 815 821 12879453 10.1002/bies.10313

[B103] Gould SJ Vrba I Exaptation-a missing term in the science of form Paleobiology 2003 8 4 15

[B104] Jurka J Sequence patterns indicate an enzymatic involvement in integration of mammalian retroposons Proc Natl Acad Sci USA 1997 94 5 1872 1877 9050872 10.1073/pnas.94.5.1872 PMC20010

